# Construction of nomogram model for early death risk in patients with severe traumatic brain injury

**DOI:** 10.3389/fneur.2025.1683355

**Published:** 2026-01-09

**Authors:** Gang Xu, Guizhi Chen, Libin Zheng, Luqiao Xu, Yiqun Wang

**Affiliations:** Emergency Intensive Care Unit, Yongkang First People's Hospital, Jinhua, China

**Keywords:** nomogram, brain injuries, Glasgow Coma Scale, traumatic brain injury, intracranial pressure

## Abstract

**Background:**

Traumatic brain injury (TBI) poses significant challenges in prognostication and clinical management, particularly in severe cases. The need for precise prognostic tools to predict outcomes, including early death, in severe TBI is crucial. This study aimed to construct a nomogram model for early death risk in patients with severe TBI to enhance clinical decision-making.

**Methods:**

This retrospective cohort study included severe TBI patients categorized into non-survivors and survivors groups from August 2018 to March 2024. Data on demographic, clinical, laboratory, and imaging parameters were collected and analyzed by using SPSS 29.0 statistical software. A nomogram model was constructed. The model’s predictive performance was assessed using the Hosmer-Lemeshow goodness-of-fit test and ROC curve.

**Results:**

Lower Glasgow Coma Scale (GCS) scores, elevated neutrophil-to-lymphocyte ratio (NLR), prolonged prothrombin time, increased midline shift, and higher C-reactive protein (CRP) levels were associated with poor prognostic outcomes (*p* < 0.05). The collaborative nomogram model demonstrated an area under the ROC curve of 0.956, signifying its high predictive value for early death risk in severe TBI.

**Conclusion:**

The study identified several prognostic indicators, including clinical, laboratory, and imaging parameters, associated with early death risk in severe TBI patients. The constructed nomogram model offers a comprehensive tool for predicting early death risk, facilitating individualized prognostication and informed decision-making.

## Introduction

Traumatic brain injury (TBI) represents a substantial public health concern, with an estimated annual incidence of 69 million individuals affected worldwide (1). However, this global burden is characterized by significant heterogeneity in case mix, injury mechanisms, clinical management, and outcomes, particularly between high-income countries (HICs) and low- and middle-income countries (LMICs). A recent prospective global collaborative study (the Global Neurotrauma Outcomes Study) highlighted these disparities, reporting considerable variations in the management and mortality of patients undergoing emergency neurosurgery for TBI across different regions of the world (2). Among these cases, severe TBI accounts for a significant proportion, posing a considerable burden on healthcare systems both in prognostication and clinical management. Severe TBI presents complex clinical scenarios due to the potential for significant neurological impairment and a high risk of mortality (3, 4). Unlike mild or moderate TBIs, which may have a relatively favorable prognosis, severe TBI often leads to profound and long-lasting consequences, impacting not only the individual’s quality of life but also posing substantial challenges for healthcare professionals and healthcare systems (5, 6). In cases of severe TBI, the primary focus of medical care was to mitigate secondary brain injury while providing optimal support for the injured brain (7). However, due to the critical nature of these injuries, treatment decisions must be informed by precise prognostic tools that accurately assess the severity of the injury and predict the likelihood of various outcomes, including early death (8–10). Given the high stakes involved, including the potential for long-term disability or loss of life, the need for robust prognostic tools to guide treatment decisions and care planning was paramount (11–13). Early posttraumatic seizures were related to longer ICU and hospital admissions, ICU ventilation, and poorer one-year outcomes including mortality and development of PTE (14). While numerous prognostic indicators have been explored in the context of severe TBI, the construction of a comprehensive and visually accessible predictive model remains a pressing need. In this retrospective cohort study, we aimed to construct a nomogram model for early death risk in patients with severe TBI and identify potential prognostic indicators to enhance clinical decision-making.

## Materials and methods

### Study design and population

A retrospective study was conducted on severe TBI patients from August 2018 to March 2024 in our hospital. This study has obtained approval from the Institutional Review Board and Ethics Committee of Yongkang First People’s Hospital. The study was conducted in accordance with ethical guidelines. Informed consent for this retrospective study was waived as only de-identified patient data were utilized, posing no potential harm or impact on patient care. This waiver was approved by the institutional review board and ethics committee of our institution in accordance with regulatory and ethical guidelines pertaining to retrospective studies. We included patients with the following conditions: Definite history of head injury with a clinical diagnosis of severe TBI (15, 16); admission Glasgow Coma Scale (GCS) score ≤ 8; Age ≥ 16 years; Admission to the intensive care unit (ICU) due to the severity of the condition; Hospital admission and surgical treatment within 24 h of injury; Meeting surgical indications, with all surgeries performed by the same group of physicians; Normal mental and cognitive function; Complete patient records. Moreover, patients were excluded if any of the following conditions were present: patients undergoing cardiopulmonary resuscitation; concomitant severe polytrauma; severe infection, shock, significant liver or kidney dysfunction, and malignancy; repeated multiple blood transfusions; time from injury to surgery ≥ 5 h; concomitant severe hypertension and/or diabetes; history of previous cranial surgery and intracranial tumors.

### Treatment methods

Upon admission, all patients received immediate establishment of intravenous access, wound debridement, and, based on their condition, tracheal intubation and respiratory support. For those with severe head injuries, initial management included rapid assessment and stabilization of the airway, breathing, and circulation (ABCs). Patients with significant intracranial injuries were managed according to advanced trauma life support (ATLS) protocols, which included monitoring intracranial pressure (ICP) and implementing measures to control ICP and maintain cerebral perfusion pressure (CPP). For those requiring ICP monitoring, we utilized an intraparenchymal ICP monitor (Codman MicroSensor, Johnson & Johnson, USA) which was placed in the right frontal lobe. The decision to place the ICP monitor was based on clinical judgment and the presence of mass lesions or signs of increased ICP. Surgical treatment was administered according to surgical indications (17, 18), which included decompressive craniectomy, hematoma evacuation, and management of ICP. Based on the patients’ survival within 30 days post-surgery, they were categorized for prognosis. Patients who showed improvement in their condition (symptoms and CT impact report were better than before) and had stable vital signs (normal respiration, heart rate, blood pressure, and temperature), leading to transfer to general wards or discharge, were included in the survivors group. Patients who died within 30 days post-surgery were included in the deceded group. All patients included in the study underwent surgical treatment within 24 h of injury, as per our inclusion criteria, ensuring that all participants received the same initial management approach.

### Diagnostic indicators

Patient demographic data was retrieved through systematic case searches. Upon admission, the Glasgow Coma Scale (GCS) was utilized to assess the level of consciousness, consisting of eye response (0–4 points), verbal response (0–5 points), and motor response (1–6 points). Scores of 3–8 indicated severe coma, 9–12 indicated moderate coma, and 13–15 indicated mild coma. The maximum GCS score was 15, with lower scores indicating more severe consciousness impairment. The Cronbach’s *α* coefficient for the scale was 0.85. Blood samples were collected from the patients’ fasting cubital veins, mixed, and anticoagulated before subjecting them to various analyses, including prothrombin time (PT) using an Automated coagulation analyzer (CS5100, SYSMEX, Japan), and white blood cell count, hemoglobin, platelet count, and lymphocyte count using an Automatic Blood Cell Counter Plus Crp (BC-7500, Mindray, China). C-reactive protein (CRP) levels were measured by enzyme-linked immunosorbent assay (ELISA), and neutrophil count was determined using an Automatic Blood Cell Counter Plus Crp (BC-7500, Mindray, China), with the lymphocyte count/neutrophil count ratio calculated. After treatment, patients underwent head CT scans to measure midline shift. Midline shift in CT images refers to the deviation of the midline structure of the brain (such as falx cerebri, septum pellucidum, third ventricle, etc.) from the normal position. A predictive model was established based on the results of multi-factor logistic regression analysis, and R software 4.2.0 and related programs were used to construct column line risk prediction models. The predictive performance of the column line risk prediction model was assessed using the Hosmer-Lemeshow goodness-of-fit test and receiver operating characteristic (ROC) curve analysis, with the area under the curve (AUC) calculated. Early death was defined as death within 1 month after the onset of brain injury.

### Statistical analysis

Data analysis was conducted using SPSS 29.0 statistical software (SPSS Inc., Chicago, IL, USA). Categorical data were expressed as [*n* (%)] and tested using the chi-square test with the basic formula when the sample size was ≥ 40 and theoretical frequency T ≥ 5. When the sample size was ≥ 40 but the theoretical frequency was 1 ≤ T < 5, the chi-square test was performed using the corrected formula. For sample sizes < 40 or theoretical frequency < 1, statistical analysis was done using Fisher’s exact probability method. Normality of continuous variables was assessed using the Shapiro–Wilk test. For normally distributed continuous variables, results were presented as mean ± standard deviation (SD) and analyzed using the t-test with corrected variance. Non-normally distributed data were presented in the form of median (quartile) and analyzed using the Wilcoxon rank-sum test. A two-tailed *p* < 0.05 was considered statistically significant. Pearson correlation analysis was used for continuous variables, and Spearman correlation analysis was used for categorical variables. Prior to constructing the multivariable logistic regression model, multicollinearity among the candidate predictor variables was assessed using the variance inflation factor (VIF). A VIF value of < 5 was considered to indicate no significant multicollinearity.

## Results

### Baseline characteristics

Based on prognostic outcomes, the patients categorized into non-survivors group (*n* = 153) and survivors group (*n* = 151). Comparison of baseline characteristics between patients with severe TBI in the two groups, several key findings were observed (Table 1). The age and gender difference between the two groups was not statistically significant (*p* > 0.05). In contrast, the GCS score exhibited significant differences between the two groups (*p* < 0.05). These findings lay the groundwork for the construction of a nomogram model for early death risk in patients with severe TBI, with the identified variables serving as potential predictive factors.

**Table 1:** Baseline characteristics of non-survivors and survivors groups in patients with severe TBI.

Parameter	Non-survivors group (*n* = 153)	Survivors group (*n* = 151)	t/χ^2^/W	*p* value
Age (years, mean ± SD)	57.45 ± 17.24	53.06 ± 18.89	0.969	0.336
Gender (*n*, %)				
Male	46 (30.07%)	54 (35.76%)	1.117	0.291
Female	107 (69.93%)	97 (64.24%)		
GCS score (mean ± SD)	4.30 ± 1.26	6.10 ± 1.19	25.681	< 0.001

### Laboratory findings

In comparing the laboratory findings between patients with severe TBI in the non-survivors and survivors groups, several noteworthy observations were made (Table 2). Hemoglobin levels did not demonstrate statistical significance between the two groups. However, platelet count, lymphocyte count and neutrophil-to-lymphocyte ratio (NLR) displayed a significant difference. Furthermore, the PT was notably prolonged in the deceded group relative to the survivors group (*p* = 0.029). White blood cell count and neutrophil count, on the other hand, did not exhibit a significant difference between the two groups. The CRP levels were markedly higher in the non-survivors group compared to the survivors group (*p* < 0.05). These findings contribute to the development of a nomogram model for evaluating early death risk in patients with severe TBI, underscoring the potential utility of these laboratory parameters as prognostic indicators.

**Table 2:** Laboratory findings of non-survivors and survivors groups in patients with severe TBI (mean ± SD, median).

Parameter	Non-survivors group (*n* = 153)	Survivors group (*n* = 151)	t/W	*p* value
Platelet count (10^9^/L)	153 (75, 174)	174 (117.25, 226.5)	674	0.029
Neutrophil count (10^9^/L)	14.16 ± 6.88	14.44 ± 5.10	0.191	0.849
Lymphocyte count (10^9^/L)	1.04 (0.67, 1.42)	1.18 (1.12, 1.74)	676	0.028
White blood cell count (10^9^/L)	16.75 ± 8.18	17.53 ± 5.64	0.446	0.657
Hemoglobin (g/L)	105.30 ± 25.77	113.87 ± 18.03	1.549	0.127
NLR	13.82 ± 4.47	10.96 ± 3.88	2.743	0.008
Prothrombin time (s)	15.1 (13.7, 18.1)	13.5 (12.9, 14.4)	300.5	0.005
CRP (mg/L)	121.41 ± 53.81	72.26 ± 40.30	4.152	< 0.001

Since platelet count, lymphocyte count, and prothrombin time are non-normally distributed data, they are presented in the table as median (interquartile range).

### Imaging findings

**Figure 1:**
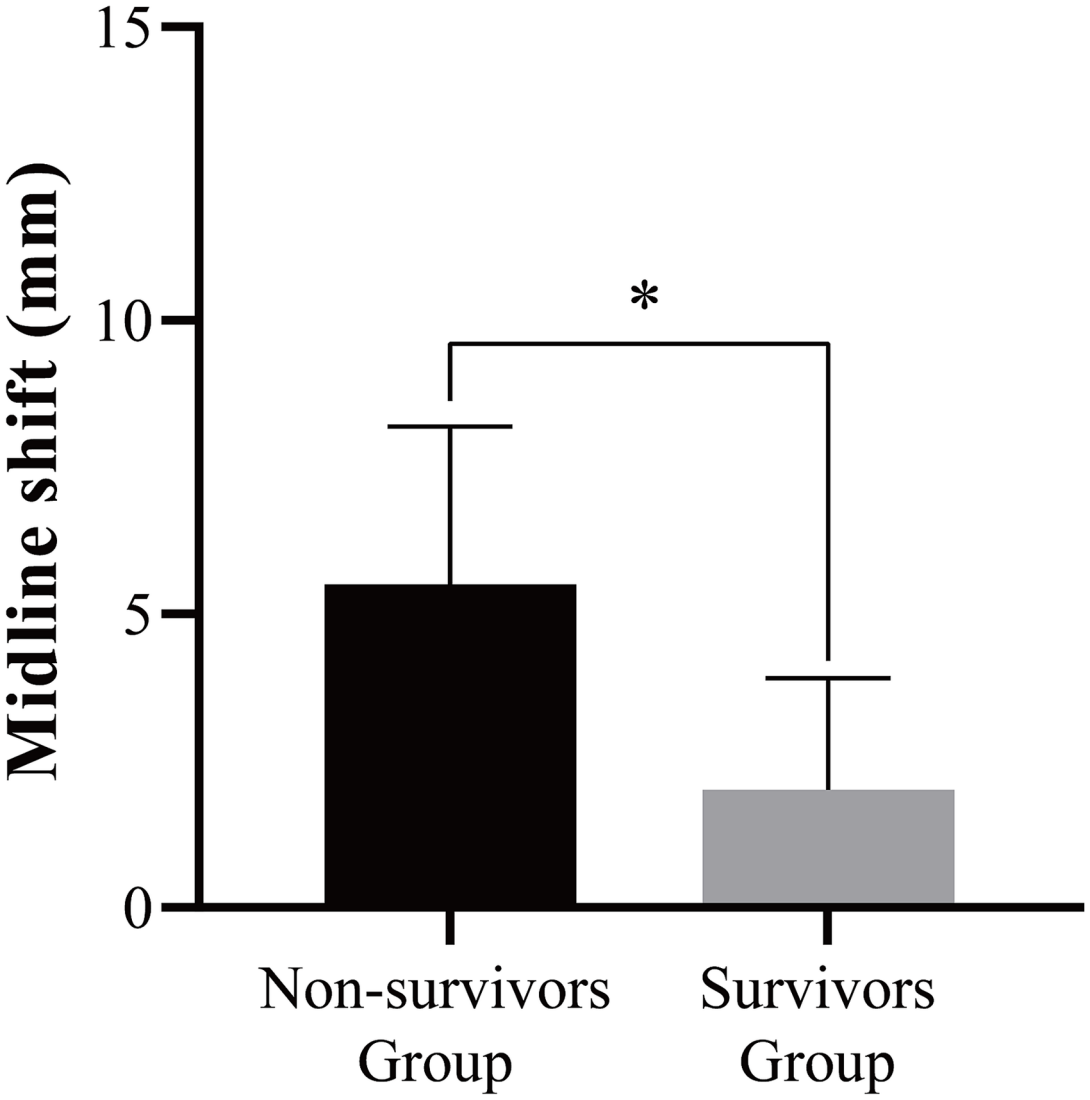
Imaging findings of non-survivors and survivors groups in patients with severe TBI. *p < 0.05.

In comparing the imaging findings between patients with severe TBI in the non-survivors and survivors groups, a statistically significant difference was observed in midline shift (Figure 1). This disparity in midline shift between the two groups underscores its potential value as a prognostic indicator for early death risk in patients with severe TBI.

### Correlation analysis

In conducting correlation analyses of adverse prognosis in patients with severe TBI using various indicators, several significant findings were revealed. The GCS score exhibited a strong positive correlation with adverse prognosis (*p* < 0.001), indicating its potential as a prognostic indicator (Table 3). Conversely, lymphocyte count, NLR, platelet count, PT, and midline shift exhibited statistically significant but comparatively weaker correlations with adverse prognosis. Notably, CRP levels demonstrated a strong negative correlation with adverse prognosis (*p* < 0.001), underscoring its potential as a valuable prognostic indicator for early death risk in patients with severe TBI. These correlation analyses contribute to the construction of a nomogram for evaluating early death risk in this patient population.

**Table 3:** Correlation analysis of adverse prognosis in patients with severe TBI using various indicators.

Parameter	r	R^2^	*p* value
GCS score	0.595	0.354	< 0.001
Platelet count (10^9^/L)	0.295	0.087	0.018
Lymphocyte count (10^9^/L)	0.269	0.072	0.032
NLR	−0.328	0.107	0.008
Prothrombin time (s)	−0.377	0.142	0.002
CRP (mg/L)	−0.463	0.214	< 0.001
Midline shift (mm)	−0.315	0.099	0.011

### Construction of the nomogram

**Figure 2:**
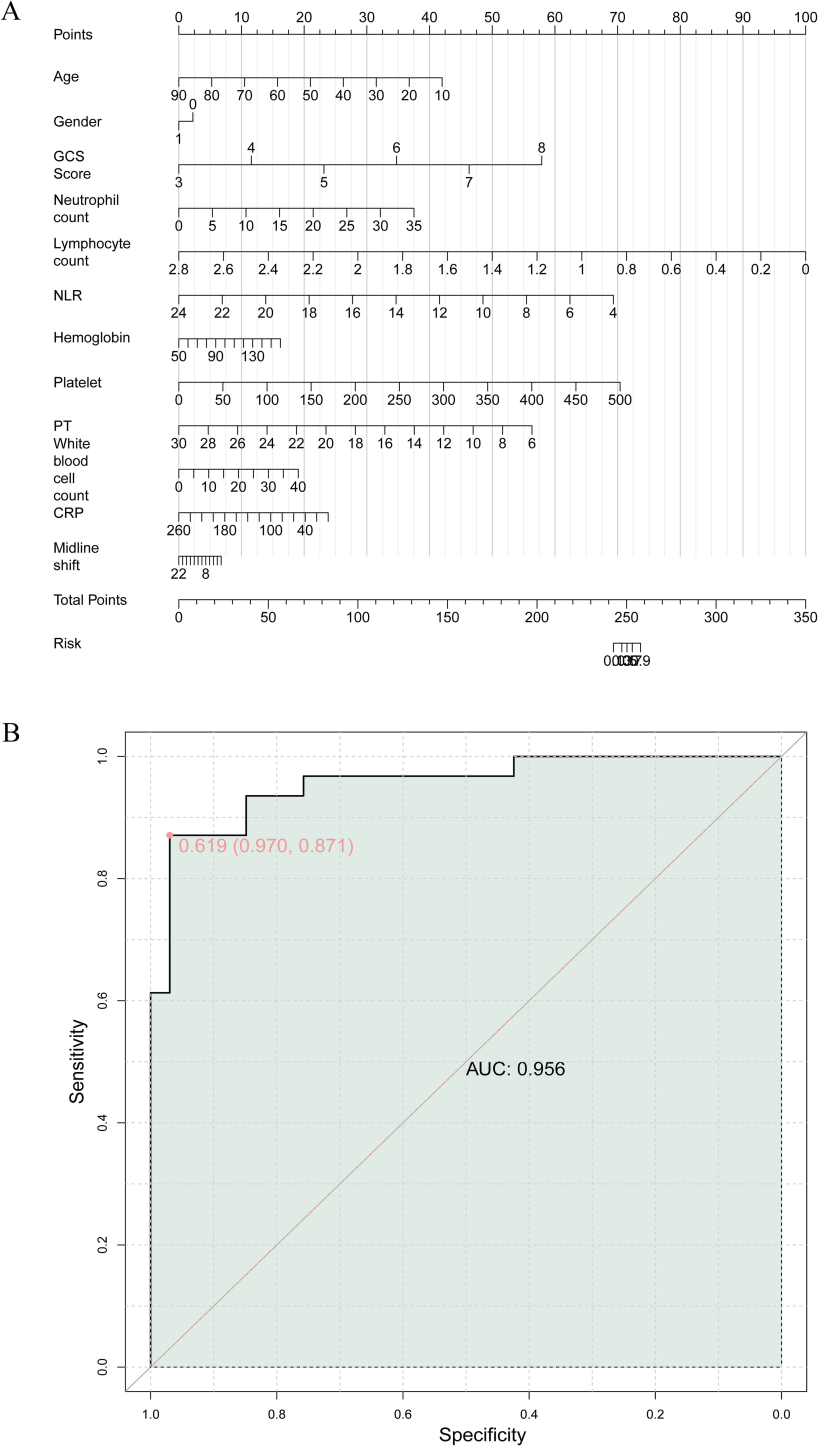
**(A)** Construction of a nomogram model of early death risk in patients with severe craniocerebral injury. **(B)** The receiver operating characteristics (ROC) curve and area under the curve (AUC) of early death risk in patients with severe craniocerebral injury.

Before developing the multivariable model, we assessed the potential multicollinearity among all included predictors, including GCS, NLR, PT, CRP, and midline shift. The variance inflation factor (VIF) for each variable was well below the threshold of 5 (all VIFs < 2.0), indicating that severe multicollinearity was not present and that the parameter estimates in our logistic regression model are stable and reliable. Based on the results of multifactor analysis, a risk prediction model was presented in the form of a column line chart. Finally, this study combined indicators with predictive value to construct a joint model for predicting early death risk in patients with severe TBI (Figure 2A). ROC curve was that the AUC was a valuable metric for evaluating the diagnostic efficacy of the classification model and an AUC value of 0.9 or higher is indicative of excellent performance. The results of the ROC analysis showed an AUC value of 0.956, indicating that the joint model had extremely high predictive value for early death risk in patients with severe TBI (Figure 2B).

## Discussion

TBI was a significant public health concern, with severe cases resulting in substantial morbidity and mortality (19). Early prognostication of patient outcomes in severe TBI was crucial for guiding clinical management and resource allocation (20, 21). In this retrospective cohort study, we sought to construct a nomogram model for early death risk in patients with severe TBI and identify potential prognostic indicators to enhance clinical decision-making. Our study identified several key factors associated with early death risk in severe TBI patients. We found that the Glasgow Coma Scale (GCS) score, laboratory parameters, and imaging findings are key factors associated with early death risk. The GCS score, a fundamental tool for assessing consciousness following TBI, exhibited a strong positive correlation with adverse prognosis, consistent with previous literature (22, 23). Lower GCS scores were significantly associated with poor prognostic outcomes, highlighting its utility as a prognostic indicator for early death risk in severe TBI patients. The GCS score has been widely recognized as a crucial factor in determining the severity of TBI and was commonly utilized in prognostic models and clinical decision-making algorithms (24–27). Our findings further emphasize the importance of prompt and accurate GCS assessment in the initial evaluation of severe TBI patients.

In addition to the GCS score, laboratory parameters such as neutrophil count, lymphocyte count, NLR, and CRP levels were found to be associated with early death risk in severe TBI patients. Elevated levels of NLR and CRP were significantly associated with adverse prognostic outcomes, consistent with the findings of Nguyen A et al. and Hosseininejad SM et al. (28, 29), supporting the role of systemic inflammatory responses and immune dysregulation in the pathophysiology of TBI. The identification of these laboratory parameters as prognostic indicators provides valuable insights into the potential mechanisms underlying early death risk in severe TBI and reinforces the importance of systemic inflammatory response assessment in TBI management.

Furthermore, our study revealed imaging findings, including midline shift, as a significant prognostic indicator for early death risk in severe TBI patients. Midline shift, an indicator of intracranial mass effect, was notably associated with adverse prognostic outcomes, underscoring its value as a radiological marker in predicting patient outcomes. These findings align with previous studies (30–32) demonstrating the prognostic significance of midline shift in TBI patients and highlight the critical role of neuroimaging in risk stratification and prognostication in severe TBI. The construction of a nomogram model for early death risk in severe TBI patients constitutes a significant contribution of this study. The nomogram model, encompassing multiple prognostic indicators, provides a comprehensive and visually accessible tool for predicting early death risk in severe TBI patients. The high AUC value of 0.956 for the joint model underscores its robust predictive value and potential clinical utility. The nomogram model offers a practical approach for risk stratification and individualized prognostication (33, 34), facilitating informed decision-making and personalized care planning for severe TBI patients, which is consistent with the research results of Lang L (35).

It was important to acknowledge several limitations of this study. First, the retrospective nature of the study may introduce inherent biases (e.g., data quality, missing data) and limit the generalizability of the findings. Prospective studies with larger patient cohorts were warranted to validate the prognostic significance of the identified indicators and the nomogram model. Secondly, while our nomogram incorporates key clinical, laboratory, and imaging variables, we acknowledge that other promising prognostic markers exist. For instance, early hyperglycemia has been consistently linked to increased mortality in TBI patients, potentially reflecting the severity of the initial stress response and its exacerbating effect on secondary brain injury (36). Similarly, the De Ritis ratio (aspartate aminotransferase to alanine aminotransferase ratio), a marker of systemic physiological stress and potential hepatic hypoperfusion, has recently been identified as an independent prognostic factor for mortality in moderate-to-severe TBI (37). Although these variables were not included in our final model, their established prognostic value underscores the multifactorial nature of TBI outcomes. Future iterations of our model could benefit from exploring the additive predictive value of incorporating these and other novel biomarkers to further enhance its accuracy and comprehensiveness. Thirdly, the exclusion of patients with significant chronic comorbidities maight limit the generalizability of our findings. These subgroups represent a substantial proportion of real-world severe TBI populations, particularly among the elderly. While their exclusion was necessary to minimize confounding effects and homogenize the study population for clearer identification of intrinsic prognostic factors, it may reduce the model’s direct applicability to all-comer TBI cohorts in clinical practice. Future studies specifically including and stratifying these high-risk subgroups are warranted to validate and potentially refine the nomogram for broader use. Lastly, although the nomogram demonstrated excellent discriminative ability with an AUC of 0.956 in our cohort, this model was not subjected to internal validation techniques, such as bootstrapping or cross-validation. The reported performance metrics may therefore be subject to a degree of over-optimism, as they reflect performance on the same dataset from which the model was derived. Thus, the performance of our nomogram requires confirmation in an independent patient cohort. The next essential step for this research is to perform both rigorous internal validation and external validation in a multi-center setting to truly ascertain its generalizability and clinical utility.

## Conclusion

In conclusion, the study identified several prognostic indicators, including clinical, laboratory, and imaging parameters, associated with early death risk in severe TBI patients. The constructed nomogram model offers a comprehensive tool for predicting early death risk, facilitating individualized prognostication and informed decision-making. This nomogram can assist clinicians in assessing risk and tailoring treatment approaches to individual patient needs. While the study elucidates valuable insights, further prospective research endeavors were essential to validate and expand upon the findings, ultimately enhancing the management and outcomes of severe TBI patients.

## Data Availability

The original contributions presented in the study are included in the article/supplementary material, further inquiries can be directed to the corresponding author.
